# Allostery and Epistasis: Emergent Properties of Anisotropic Networks

**DOI:** 10.3390/e22060667

**Published:** 2020-06-16

**Authors:** Paul Campitelli, S. Banu Ozkan

**Affiliations:** Department of Physics and Center for Biological Physics, Arizona State University, Tempe, AZ 85287, USA; Paul.Campitelli@asu.edu

**Keywords:** epistasis, allostery, elastic network model, protein conformational dynamics

## Abstract

Understanding the underlying mechanisms behind protein allostery and non-additivity of substitution outcomes (i.e., epistasis) is critical when attempting to predict the functional impact of mutations, particularly at non-conserved sites. In an effort to model these two biological properties, we extend the framework of our metric to calculate dynamic coupling between residues, the Dynamic Coupling Index (DCI) to two new metrics: (i) EpiScore, which quantifies the difference between the residue fluctuation response of a functional site when two other positions are perturbed with random Brownian kicks simultaneously versus individually to capture the degree of cooperativity of these two other positions in modulating the dynamics of the functional site and (ii) DCI_asym_, which measures the degree of asymmetry between the residue fluctuation response of two sites when one or the other is perturbed with a random force. Applied to four independent systems, we successfully show that EpiScore and DCI_asym_ can capture important biophysical properties in dual mutant substitution outcomes. We propose that allosteric regulation and the mechanisms underlying non-additive amino acid substitution outcomes (i.e., epistasis) can be understood as emergent properties of an anisotropic network of interactions where the inclusion of the full network of interactions is critical for accurate modeling. Consequently, mutations which drive towards a new function may require a fine balance between functional site asymmetry and strength of dynamic coupling with the functional sites. These two tools will provide mechanistic insight into both understanding and predicting the outcome of dual mutations.

## 1. Introduction

A growing body of data on the human genome suggest that within the exome (the protein coding region), one individual may possess 10,000 or more non-synonymous nucleotide variants, many of which occur at positions which are not evolutionarily conserved [1,2,3]. Predicting the functional outcome of mutations at non-conserved sites remains an extremely difficult challenge. In particular, providing accurate predictions about the impact of these variations is difficult when only considering single, independent point mutations without accounting for the background of other positions and their chemical specificity (i.e., context dependence).

One reason why predicting the impact of mutations may fail is that extensive epistasis occurs during evolution [4,5,6]. Epistasis is defined as a context-dependent functional outcome, where, the alternative context could be just one single amino acid difference, or it could be a paralog with 25% sequence identity. Experimentally, epistasis manifests as a non-additive outcome from two or more amino acid changes within a protein. The effects can be dramatic. For example, a substitution may only confer a beneficial effect upon fixation of a second-site, also known as a “permissive” change; conversely, a neutral substitution might become deleterious in the presence of other “restrictive” substitutions [7,8,9]. Thus, epistasis plays a vital role in shaping trajectories of protein evolution [4,5,6,7,8,9,10]. Furthermore, mounting evidence indicates that protein evolution, particularly evolution towards new function, proceeds not only through mutations at functionally critical sites, but also through sites which can have a subtle (or, occasionally, substantial) effect on function when mutated without being immediately identifiable as positions with particular functional or structural importance [11,12,13].

Epistatic relationships becomes crucial when comparing homologous protein families or protein domains, which can exhibit significant sequence variation and biochemical properties that may span orders of magnitude while still maintaining a similar three-dimensional (3-D) fold [14,15,16,17,18,19]. Thus, single or dual mutations on homologous proteins yield a wide range of functional outcomes [20,21,22,23,24]. In fact, understanding the mechanics or predicting the results of dual mutations remains a significant challenge in the presence of systems which experience large epistatic effects, even when accurate experimental data are available for the single mutant systems [7,8,9,10,25].

On the other hand, when protein equilibrium dynamics and each individual position’s contribution to these dynamics are taken into consideration, we can shed light onto the mechanism of epistatic relations. This is because proteins sample many different conformations within the native state, and these conformational dynamics, governed by the strength of the 3-D network of interactions, underlie protein function. Within this dynamic view, we can simply treat a protein as a biological signal processor where the 3-D interaction network mediates long-range communication through amino acid fluctuations nascent to a given protein sequence. Therefore, the knowledge of how mutations may fine-tune this sequence-function relationship necessitates evaluating the role of each residue position in establishing a protein’s internal communication network through protein dynamics [26,27]. Particularly, when two substitutional sites are considered together, the dynamic coupling of these sites results in a joint effect (i.e., a cooperative response) leading to the modulation of signal processing responsible for biophysical behavior and, ultimately, may give rise to a non-additive functional outcome.

The non-additive, epistatic interactions therefore can use dynamic features of a protein to modulate function. These dynamics features are similar to that found in allosteric modulation in which a protein is able to control catalytic function or regulate on/off states through the binding of a ligand to a site distal from a catalytic/active site. This distal binding has been shown to modulate catalytic site dynamics, sometimes without association to distinct conformational states. This type of allostery, which can impact function by manipulating the normal modes of the protein while retaining the conformation, is known as dynamic allostery [28,29]. We now understand this form of allosteric regulation to be a specific, and often more dramatic, emergent property of the unique internal networking between amino acids within a protein. To this end, allosteric systems reduce the enormous dimensionality associated with information transfer and communication pathways for these complex, anisotropic networks by identifying important regulation sites a priori. Therefore, as observed in allosteric regulations, the long-distance interactions through dynamic coupling between different positions and active sites can be modulated and re-wired through substitutions, which emerge as epistasis that drives the evolution of new function. Here, we aim to identify these epistatic relations through the development of dynamics-based metrics which can measure the strength of long-range dynamic interactions.

The modeling of protein conformational dynamics using force perturbations and elastic networks has been previously used successfully in attempts to understand the role of long-range interactions in protein evolution [30,31,32,33]. Here we attempt to model these effects through the use of Perturbation Response Scanning (PRS) and the Elastic Network Model (ENM) to construct a Dynamic Coupling Index (DCI) where we can capture the dynamic coupling between any given residue pair or set of residues via a system’s response to random force perturbations. DCI captures the strength of displacement response of a given position *i* upon perturbation to a single position (or subset of positions) *j*, relative to the average fluctuation response of position *i* when all of the positions within a structure are perturbed. Expanding upon the dynamic coupling concept, here we develop a new metric called EpiScore. EpiScore measures the difference in the residue fluctuation response of an active site when two mutational sites are simultaneously perturbed by random forces versus the response when individual force perturbations are exerted one at a time to the mutational sites.

In order to determine whether EpiScore can identify the degree and strength of epistatic relationships between position pairs, we first applied our analysis to the deep scanning database of double mutations between all positions in the IgG-binding domain of protein GB1 [24]. These modern, high-throughput screens (e.g., deep mutational scans) assay large numbers of mutants (up to 10^8^) [34,35,36,37], but the information is largely qualitative. Therefore, to further test whether our approach can identify epistatic relations which specifically emerge during the evolution of new function, we applied our methodology to two different protein systems where the traditional biochemical quantifications of mutational effects (e.g., k_cat_, K_M_, IC_50_) for a range of substrates are available. These two systems, *P. falciparum* DHFR (pfDHFR) and a β-lactamase (TEM-1), naturally confer resistance to drugs and the trajectories of these resistances as well as their epistatic relationships have been explored [22,23]. Importantly, these two systems are also known to be allosteric proteins.

We first observed that EpiScore can distinguish positive and negative epistasis in dual mutations when analysis was performed over 1045 single mutants and 509,963 double mutants of GB1. We also found that the average EpiScore value correlates well with experimental epistatic measures calculated using pyrimethamine IC_50_ values of pfDHFR dual mutants and the catalytic turnover rates for cefotaxime of TEM-1 dual mutants. Furthermore, each pfDHFR amino acid pair exhibits distinct distributions of EpiScore values showing the importance of how these two positions communicate with the active site through the anisotropic interaction network.

Interestingly, DCI is usually not symmetric, i.e., the fluctuation response of position *i* upon exerting random forces on *j* is not identical to the response of *j* when position *i* is perturbed; we calculate this asymmetry with DCI_asym_. We applied our DCI_asym_ analysis to the TEM-1 dual-mutant sites and found that, indeed, a relationship exists between dynamic coupling asymmetry and EpiScore when all active sites in the TEM-1 system are considered. Specifically, two of the three dual mutant positions which exhibited the largest positive epistasis in cefotaxime k_cat_/K_M_ from the wild-type had both EpiScore values < 1 (indicating strong non-additivity) with respect to active site S70. Additionally, these dual mutants also exhibit asymmetry in dynamic coupling based on DCI_asym_, with consistent unidirectionality from active sites site to mutation sites in long range communication. We propose that this communication directionality signature should be readily apparent in known allosteric systems as mentioned above. Therefore, we applied a similar analysis to a Pin1 protein well-studied for its dynamic allostery and showed that the DCI_asym_ between the catalytic binding sites and non-catalytic distal binding sites presents a unique directionality in long distance dynamic coupling, leading to a cause-and-effect relationship between allosteric sites and active sites also observed in epistatic interactions.

## 2. Methods

We previously designed a unique way to capture site-specific coupling between residue pairs or groups of residues, the Dynamic Coupling Index (DCI). The underlying premise behind DCI is the importance of a system’s response to a force perturbation, be that protein-solvent, protein-protein, protein-ion or protein ligand interactions. Additionally, the point mutations here are modeled by the response of a system to a perturbation at a specific site, a.k.a. a single amino acid.

**Figure 1:**
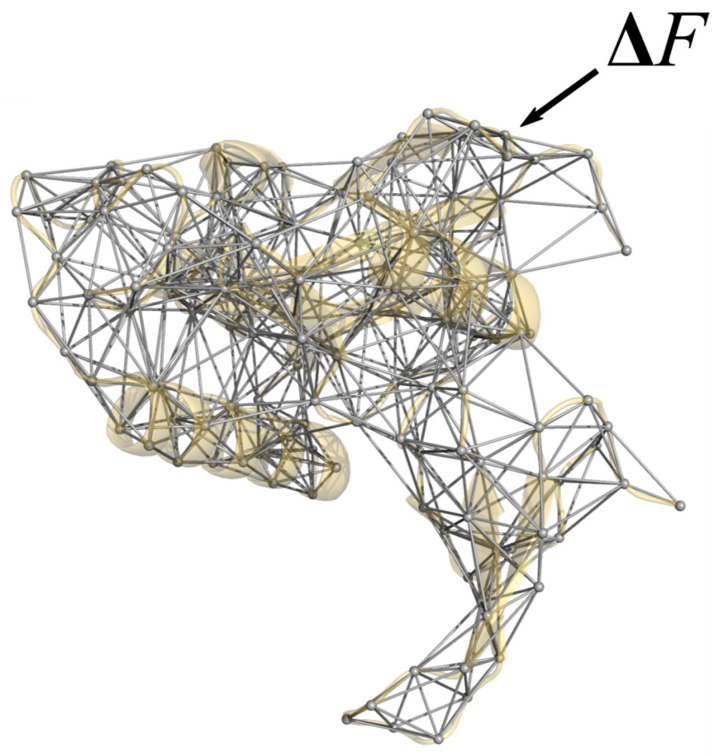
Elastic network model representation of protein Pin1 (PDB ID 3TCZ [7], ligands removed). Here, each residue within the structure is represented as a single node at the Cα position, connected to other nodes via Hookean springs. Using a combination of Perturbation Response Scanning (PRS) and Linear Response Theory (LRT) [38,39], each residue is perturbed by a Brownian kick applied as an isotropic external force which then generates a fluctuation response in all other residues within the network. This figure was rendered in PyMol [40].

DCI is a combination of the Elastic Network Model (ENM) and Linear Response Theory (LRT) where the protein is modeled by representing the amino acids as nodes in a network connected by Hookean springs (Figure 1). The interaction between two amino acids close in space due to their 3-dimensional structure is represented by a simple harmonic function. A random Brownian kick in the form of a unit force perturbation is applied to an individual position which generates a response propagating through the rest of the structure, causing other positions to respond to this perturbation through the network of interactions. Using LRT, we can calculate the fluctuation response Δ*R* (Equation (1)) of each position and create response vector that measures the magnitude and direction (x, y and z) of displacement of every residue from its mean. As mentioned above, this (to the first order) mimics the effects of in vivo interactions of a protein. For example, a ligand binding event will apply a force to residues in the binding pocket of a receptor protein. In our perturbation residue scanning (PRS) approach, this is averaged over many unit force directions to simulate an isotropic perturbation.

(1) $${\left[\Delta R\right]}_{3N\times 1}={\left[H\right]}_{3N\times 3N}^{-1}\;{\left[F\right]}_{3N\times 1}$$

**H** is the Hessian, a 3N × 3N matrix which can be constructed from 3-dimensional atomic coordinate information where it is composed of the second order derivatives of the harmonic potential energy with respect to the components of the position vector of length 3N. The Hessian matrix can be extracted directly from molecular dynamics simulations as the inverse of the covariance matrix. This method allows one to implicitly capture specific physiochemical properties and more accurate residue-residue interactions via atomistic force fields and subsequent all-atom simulation data. However, for the purposes of this paper, we wished to investigate only those relationships which could be derived solely from inter-atomic distances of single protein structures and thus we used the ENM version of our approach.

Repeating this process, each position in the structure is perturbed sequentially to generate a perturbation response matrix **A**

(2) $$A_{N\times N}=\left[\begin{array}{ccc} {\left|{\Delta R}^{1}\right|}_{1} & \cdots & {\left|{\Delta R}^{N}\right|}_{1}\\ \vdots & \ddots & \vdots \\ {\left|{\Delta R}^{1}\right|}_{N} & \cdots & {\left|{\Delta R}^{N}\right|}_{N} \end{array}\right]$$

where ${\left|{\Delta R}^{j}\right|}_{i}=\sqrt{\langle {\left(\Delta R\right)}^{2}\rangle }$ is the magnitude of fluctuation response at position *i* due to the perturbations at position *j*. From this perturbation response matrix, we can construct DCI. DCI_ij_, then, represents the displacement response of position *i* upon perturbation to a single functionally important position (or subset of positions) *j*, relative to the average fluctuation response of position *i* when all of the positions within a structure are perturbed.

(3) $${\mathrm{DCI}}_{\mathrm{ij}}=\frac{\sum_{j}^{N_{\mathrm{functional}}}{\left|{\Delta R}^{j}\right|}_{i}/N_{\mathrm{functional}}}{\sum_{j=1}^{N}{\left|{\Delta R}^{j}\right|}_{i}/N}$$

As such, DCI can be considered a measure of the dynamic coupling between residue *i* and residue(s) *j* upon perturbation to residue(s) *j.*

It is often more convenient to represent DCI as a percentile rank,

(4) $${\%\mathrm{DCI}}_{\mathrm{ij}}=\frac{m_{\leq i}}{N}$$

where m_≤i_ is the number of positions with a DCI value ≤ DCI_ij_ for a system of N residues.

One of the most important aspects of DCI is that the entire network of interactions is explicitly included in subsequent calculations without the need of dimensionality reduction such as Normal Mode Analysis through principal component analysis. If one considers interactions such as allostery as an emergent property of an anisotropic interaction network, it is critical to include the interactions of the entire network to accurately model the effect one residue can have on another.

**Figure 2:**
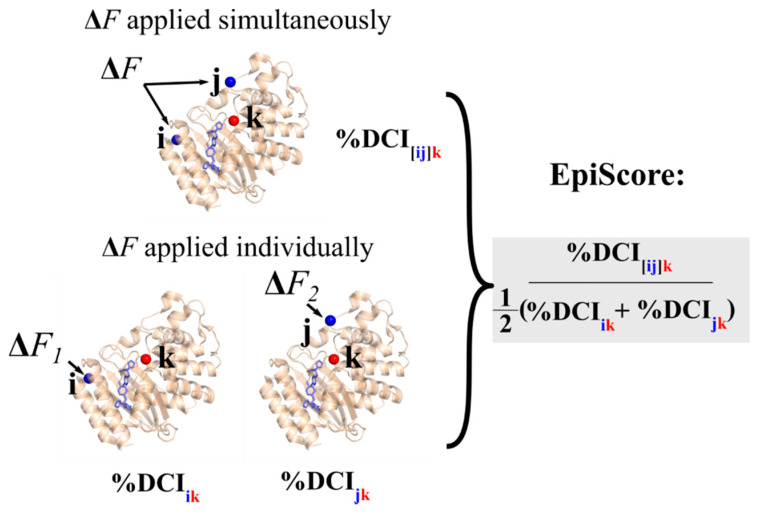
Schematic describing the calculation of EpiScore. The numerator is the %Dynamic Coupling Index (%DCI) value at position *k* upon a simultaneous perturbation to positions *i* and *j* divided by the average %DCI value at position *k* when positions *i* and *j* are perturbed individually. Thus, an EpiScore value of 1 indicates a perfect coupling additivity with respect to a given position *k* in individual versus simultaneous perturbations of two positions *i* and *j.* Figures rendered in PyMol [40] using β-lactamase (TEM-1) structure 1BTL [41].

Here, we present two further extensions of DCI which allow us to uniquely model allosteric interactions and epistatic effects; EpiScore and DCI_asym_, respectively. EpiScore can identify or describe potential non-additivity in substitution behavior between residue pairs. This metric can capture the differences in a normalized perturbation response to a position *k* when a force is applied at two residues *i* and *j* simultaneously versus the average additive perturbation response when each residue *i*, *j*, is perturbed individually (Figure 2). EpiScore values < 1 (> 1) indicate that the additive perturbations of positions *i* and *j* generates a greater (lesser) response at position *k* than the effect of a simultaneous perturbation. This means that, when treated together with a simultaneous perturbation at both sites *i* and *j*, the displacement response of *k* is lower (higher) as compared to the average effect of individual perturbations to *i* and *j*, one at a time. As EpiScore is a linear scale, the further the value from 1, the greater the effect described above.

**Figure 3:**
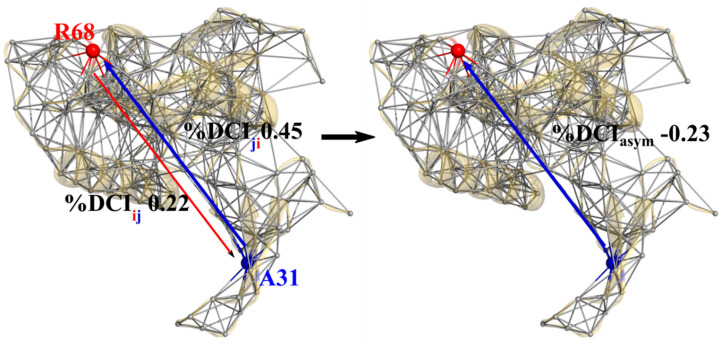
Example of asymmetric coupling between residue R68 of the PPIase domain and A31 of the WW domain in Pin1 (PDB ID 3TCZ [7]). The differences in local contacts give rise to network inhomogeneities which subsequently result in different %DCI values from R68 to A31 versus A31 to R68 (left). The subtraction of these two values gives a measure of coupling directionality upon external perturbations between these two sites (right). These figures were rendered in PyMol [40].

Interestingly, through the use of DCI we can capture asymmetry between different residues within a protein, as coupling in and of itself is asymmetric within an anisotropic network. That is, each amino acid has a set of positions to which it is highly coupled, and this anisotropy in connections gives rise to unique differences in coupling between a given *i j* pair of amino acids which do not have direct interactions (Figure 3). DCI_asym_, then, is simply DCI_ij_ (the normalized displacement response of position *j* upon a perturbation to position *i*) − DCI_ji_ (Equation (5)). Using DCI_asym_ we can determine a cause-effect relationship between the *i j* pair in terms of force/signal propagation between these two positions.

(5) $${\mathrm{DCI}}_{\mathrm{asym}}={\mathrm{DCI}}_{i}-{\mathrm{DCI}}_{j}$$

(6) $${\%\mathrm{DCI}}_{\mathrm{asym}}={\%\mathrm{DCI}}_{i}-{\%\mathrm{DCI}}_{j}$$

## 3. Results and Discussion

### 3.1. Epistasis and EpiScore

**Figure 4:**
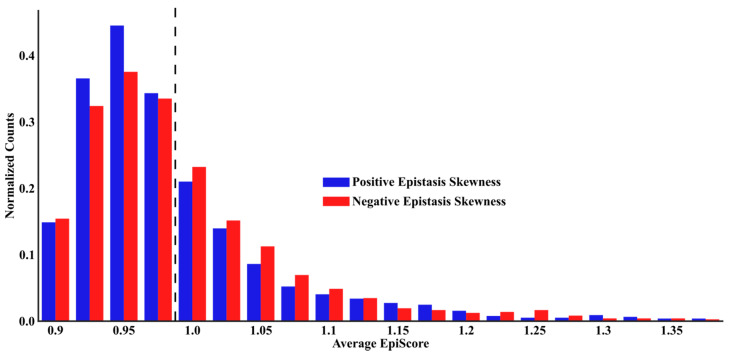
Distribution of the average EpiScore for protein GB1 protein pairs separated by positive and negative experimental epistasis using experimental deep scanning data for every position pair (excluding position 1). EpiScore values above and below one (dashed line) tend to distinctly divide cases for which experimental epistasis was more frequently skewed towards the positive (below one) and negative (above one)).

To investigate the relationship between internal networking and epistasis, we first apply our analysis to protein G domain B1 (GB1, PDB ID 2QMT [42]), for which there exists a comprehensive set of mutational data. Specifically, fitness effects of mutations were determined with high confidence for 1045 single mutants and 509,963 double mutants, with data available for all 1485 possible position pairs [24]. In this work, experimental epistasis was calculated as ln(W_ab_) − ln(W_a_) − ln(W_b_), where W_ab_ represents the fitness for the dual mutant and W_a_ and W_b_ are the fitness values for the single mutants. Here we investigate the relationship between the experimental epistasis and EpiScore by comparing the average EpiScore for each position pair with instances of positive (blue) or negative (red) epistasis using the skewness of the experimental epistasis distribution over the full mutational space available for a given pair (Figure 4). Skewness was chosen as it more accurately represented the substitution behavior than position averages, which would often tend towards zero without capturing the substitution behavior for a given position pair. EpiScore values were calculated for all position pairs relative to every other position within the protein and averaged over, generating one average EpiScore value for each pair. Interestingly, when we obtained the average EpiScore distribution of experimental positive and negative epistatic pairs we found that EpiScore values above and below one tend to distinctly divide positive from negative epistasis; positive experimental epistasis was more frequently skewed towards EpiScore > 1, and likewise negative cases are skewed towards EpiScore < 1.

**Figure 5:**
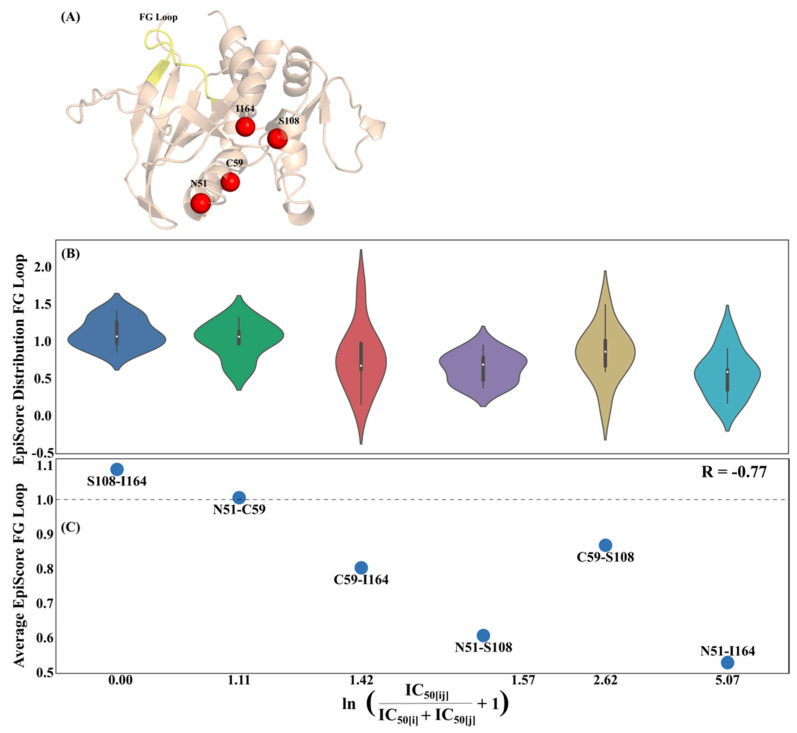
(**A**) *P. falciparum* DHFR (pfDHFR) structure (PDB ID 3QGT [48]) with FG loop residues (196–206) in yellow and mutation sites N51, C59, S108 and I164 colored in red. While not directly involved in catalytic activity, widespread use of pyrimethamine has resulted in pervasive and persistent mutations at these sites which confer pyrimethamine resistance. (**B**) Violin plot (distribution and kernel density estimate) of EpiScore values and (**C**) average EpiScore values with respect to FG loop residues for each pfDHFR dual mutant, in order of increasing pyrimethamine IC_50_ epistasis. A dashed line at EpiScore values of 1.0 has been added to aid in visual inspection. In (**B**) any FG loop residue which was within 10 angstroms of either mutation site per dual mutant was excluded from the averaging. The average EpiScore values have a strong, negative correlation (R = −0.77) with IC_50_ epistasis, where the stronger the positive epistasis, the lower the average EpiScore value.

The full system analysis of GB1 showed the existence of a general trend between epistasis and EpiScore; particularly, an inverse relationship between EpiScore above or below one and skewness in experimental epistasis, indicating that positions with EpiScore less than 1 more often work cooperatively towards beneficial protein function, whereas pairs yielding EpiScore values greater than 1 usually result in antagonistic interactions which impair function. In an effort to elucidate more specific mechanistic details or trends underlying epistatic interactions which may exist in other systems, we broaden our application of EpiScore to other known epistatic proteins with a focus on specific mutation pairs. As such, we next study DHFR, a protein involved in the development of anti-malarial resistances in malarial parasites. Anti-malarial drugs commonly target the DHFR, which catalyzes the reduction of dihydrofolate and is essential to cellular growth and proliferation. Pyrimethamine is one such drug, used to treat malaria caused by one of the most common malarial parasites, *Plasmodium falciparum,* by competitively inhibiting DHFR. While exhibiting a particularly low sequence conservation between species, most differences in sequence are from flexible loop regions [43], while the secondary structures between these loops are highly conserved across all species [44]. However, widespread use of pyrimethamine has resulted in a prevalence of pyrimethamine-resistant *P. falciparum* DHFR (pfDHFR) mutants as a result of four key amino acid substitutions at positions N51, C59, S108 and I164 which have also exhibited significant epistasis between mutation combinations [22] (Figure 5A).

An EpiScore analysis applied collectively to the behavior of the functionally important FG loop shows an immediate relationship between epistasis in pyrimethamine IC_50_ values of the pairwise mutants and their associated EpiScore values. Figure 5B shows the EpiScore violin plots (i.e., distributions and kernel density estimates) with respect to FG loop residues 196–206 for each pfDHFR mutant pair. These violin plots show that EpiScore distribution for different mutation pairs yields a different distribution for different residue pairs. S108-I164 gives a narrow distribution with a peak around 1, suggesting that force perturbations simultaneously exerted on these positions yields the same fluctuation response profile of the FG loop positions as the average of individual fluctuation responses of the FG loop when the forces are exerted individually at S108 and I164. This distribution pattern was also observed for positions N51 and C59, although at completely different positions within the protein. On the other hand, pairing the position I164 with C59 rather than with S108 results in a completely different EpiScore distribution, with diverse fluctuation responses of FG loop positions. This suggests that I164 and C59 are highly cooperative, leading to a non-additive behavior when these two positions are perturbed simultaneously. As I164 and C59 are located at different regions of the protein (Figure 5A), one can expect to observe a wide range of EpiScore values associated with this pair. This pattern tends to hold with distally located positions in the N51-S108 and C59-S108 distributions as well. Interestingly however, N51-C59 also exhibits EpiScore values less than 1, despite the fact that they belong to the same helical region. The distributions suggest that anisotropy in the network of interactions could modulate a wide range of fluctuation responses via these position pairs, which result in different functional behavior upon mutation. To determine whether the change in fluctuation response of the FG loop to simultaneous perturbations at these mutational positions can capture functional substitution outcomes, we next investigate the relationship between EpiScore and experimentally measured epistasis using pfDHFR pyrimethamine IC_50_ values.

Figure 5C presents the average EpiScore values with respect to the FG loop for each pfDHFR pairwise mutant, in order of increasing pyrimethamine IC_50_ epistasis. A dashed line at an EpiScore value of 1.0 has been added to aid in visual inspection. Here, IC_50_ epistasis is reported as the IC_50_ ratio of the dual mutant to the IC_50_ sum of the individual mutants. Any FG loop residue which was within 10 angstroms of either mutation site per dual mutant was excluded from the averaging in order to eliminate any strong dynamic coupling effects that arise as a result of direct contact interactions. The average EpiScore values have a strong, negative correlation (R = −0.77) with IC_50_ epistasis, where the stronger the positive epistasis, the lower the average EpiScore value. For example, an EpiScore value of ~0 means the pairwise dynamic coupling to FG loop positions of a dual mutant pair is negligible as compared to the average individual dynamic coupling; that is, the distal sites can individually impact position the FG loop residues allosterically. However, when treated together with a simultaneous perturbation at both sites, the displacement response of the FG loop residues are significantly lower, and, subsequently, their joint ability to allosterically regulate these FG loop positions is effectively lost. Due to the interaction network between the two distal positions with the FG loop, they may antagonistically compensate the amplitude and direction of the response when the perturbations on these two sites are exerted at the same time. To the reverse, an EpiScore value >> 1 suggests that, simultaneously, two positions may exhibit dynamic coupling to the FG loop enough such that their pairwise mutational impact fundamentally alters the role the FG loop plays within the pfDHFR interaction network resulting in loss of function.

At first, this relationship may seem somewhat counterintuitive, as one could reasonably expect that the higher the EpiScore value (i.e., the stronger the dual position dynamic coupling versus individually averaged dynamic coupling), the higher the experimental epistasis. However, when complexed with substrate, the functionally critical M20 loop [45] is stabilized in part through interactions with amino acids in the FG loop [46]. It is possible that it is more favorable, in terms of pyrimethamine resistance, to have mutations occur at position pairs that induce a smaller fluctuation response of FG loop when perturbed simultaneously, (i.e., restricting the dynamics) than the average fluctuation response of individual perturbations applied one at a time. This is in agreement with previous work which showed that point mutations to two of the FG loop amino acids in E.coli resulted in a > 30 fold decrease in the steady state hydride transfer rate constant as compared to the wild-type [47]. This could additionally explain the pervasive and persistent nature of these mutations appearing globally in pfDHFR proteins.

Expanding our study to another system important to the concept of antibiotic resistance, we analyze TEM-1, a protein which possesses antibiotic resistance largely driven by its high evolvability, with over 170 TEM-1 mutants discovered as clinical or hospital isolates [49]. TEM-1 is a well-studied enzyme in experimental or laboratory-guided evolution, in an effort to both understand the mechanisms associated with its antibiotic resistance as well as predict possible resistance-conferring mutations [49,50,51,52].

Previous work has shown that the majority of the resistance-conferring mutations in TEM-1 are both distal to (10 Å or further) and highly coupled with the active site residues [53], indicating that these mutations impact TEM-1 function by allosterically regulating active site behavior. Additionally, it is now also understood that mutations resulting in the emergence of new enzymatic function are generally destabilizing which suggests that the evolution of new function requires additional, stabilizing mutations. As such, a more complete understanding of TEM-1 mutational behavior requires an investigation into the epistatic interplay of point mutation combinations. Thus, it is an ideal system for exploration of long-range dynamic communication to understand epistatic relationships in the emergence of resistance.

**Figure 6:**
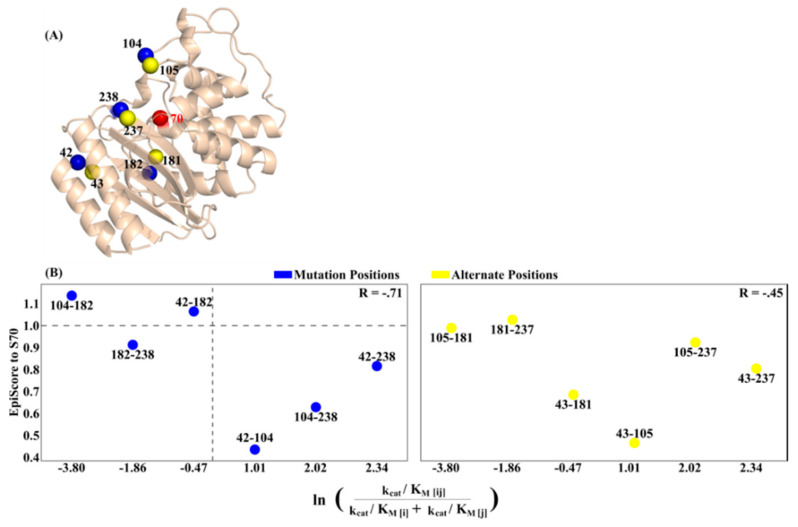
(**A**) TEM-1 structure showing mutation positions (blue spheres), alternative control positions (yellow spheres) and active site S70 (red sphere). ((**B**) left) EpiScore to active site S70 versus epistasis in ln of turnover rate of cefotaxime for β-lactamase TEM-1 mutants [23]. Horizontal dashed line divides EpiScore values above and below 1 while vertical dashed line divides positive and negative epistasis. EpiScore and epistasis exhibit a strong negative correlation of R = −0.71. EpiScore values > 1 (horizontal dashed line), indicating to a stronger pairwise dynamic coupling to position S70, also corresponds to two of the three TEM-1 dual mutants with negative epistatic turnover rates (separated by vertical dashed line). Position pair 182-238 represents a deviation from this behavior, and position pair 42-104 is a comparative outlier to the overall correlation. ((**B**) right) EpiScore versus ln of turnover rate using the alternative control positions. Although these positions are immediately adjacent to the mutation positions, they generate different EpiScore values resulting in a significantly worse correlation of R = −0.45.

Here we focus on the specific epistatic relationship between four TEM-1 mutation sites (42, 104, 182 and 238) which have exhibited significant non-additive behavior [23]. Treating point mutations as external perturbative forces to the internal network of a protein, we apply EpiScore analysis to the main TEM-1 active site, residue S70, using a TEM-1 3-D structure obtained by an energy-minimized and equilibrated version of PDB ID 1BTL [53] with mutation sites shown as blue spheres in Figure 6A, along with active site S70 in red and alternative control sites (43, 105, 181 and 237) in yellow. Figure 6B (left) shows a plot of EpiScore versus experimental epistasis using cefotaxime turnover rates and exhibits a relationship similar to that found in pfDHFR, with a strong negative correlation of R=-.71. We also find that position pairs with EpiScore values > 1.0 (horizontal dashed line), presenting a stronger pairwise dynamic coupling with position S70 compared to the average of the individual dynamic coupling, also corresponds to two of the three TEM-1 dual mutants with negative epistatic turnover rates (separated by vertical dashed line). Position pair 182-238 represents a deviation from this behavior, and position pair 42-104 is a comparative outlier to the overall correlation. The deviation of position pair 182-238 may be related to specific catalytic site interactions associated with position 238, the only position in which mutation resulted in an increase in turnover rate across all eight possible combinations of TEM-1 background. Interestingly, position 182, present in all position pairs with the three highest EpiScore values, was also the position in which mutation resulted in a significantly beneficial effect in the fewest number of possible backgrounds [23]. As a control, we also conducted this analysis using the alternative sites representing positions immediately adjacent to the four mutation sites (Figure 6B (right)). These positions result in a significantly worse correlation with cefotaxime turnover rate epistasis than the mutation positions (R = −0.45 as compared to R = −0.71), showing the sensitivity in the EpiScore metric to specific positions, regardless of separation distance.

### 3.2. Asymmetry and Epistasis

**Figure 7:**
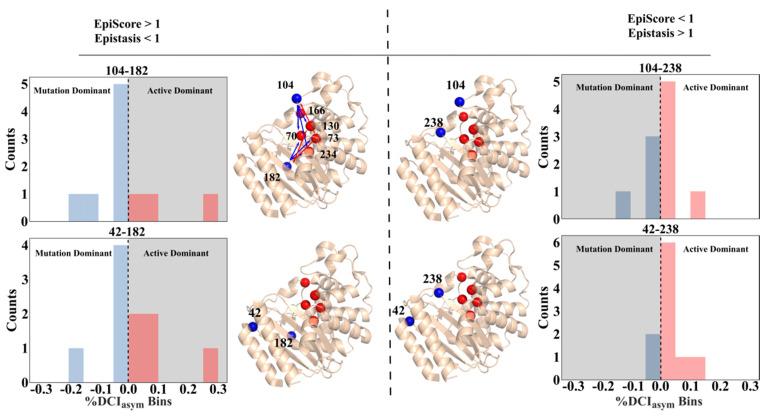
%DCI_asym_ distributions to all TEM-1 active sites (70, 73, 130, 166, 234) for each dual mutant position pair along with three-dimensional (3-D) structural representation of mutation sites (blue) and active sites (red) excluding the outliers 182-238 and 42-104 from Figure 6B. The first dual mutant (104-182, top left) has arrows drawn to indicate the coupling asymmetry, where red is active site-dominant and blue is mutation site-dominant. Both dual mutants with negative epistasis in turnover rate and EpiScore to position S70 < 1 also had %DCI_asym_ distributions which were, overall, mutation site-dominant and, conversely, those with positive epistasis in turnover rate and EpiScore to position S70 > 1 exhibited active site-dominant %DCI_asym_.

In TEM-1, mutational sites which confer incremental changes in biophysical activity are neither locally distributed with respect to one another, nor at important functional sites. Furthermore, they do not belong to an immediately identifiable allosteric inhibitor site, but they do, however, exhibit unique pairwise epistatic behavior which indicates that they likely regulate the active sites allosterically. In an effort to analyze whether the pairs having EpiScore less than 1 and associated with positive epistasis (a beneficial, cooperative interaction) exhibit long-range communication that is distinct from the pairs having EpiScore greater than 1 and associated with negative epistasis, we explored the degree of asymmetry in long-range communication between the mutational positions and the active site positions using DCI_asym_. Thus, we calculated DCI_asym_ between each TEM-1 dual-mutant site and all main active sites for the relevant TEM-1 structure (70, 73, 130, 166, 234, Figure 7), excluding the outliers 182-238 and 42-104 from Figure 6B. Here, positive %DCI_asym_ values indicate active- site-dominant dynamic coupling, where mutational sites exhibit higher fluctuation response when the active site is perturbed. On the other hand, negative %DCI_asym_ values indicate mutation-dominant dynamic coupling where perturbations at those positions controls the active site fluctuation response. Interestingly, we observe a relationship that provides some mechanical insight relating the degree of asymmetry to EpiScore; the dual mutants with EpiScore > 1 to active site S70 and epistasis < 1 had more instances of mutational-dominant coupling asymmetry, while the reverse was true for two of the three dual mutants with EpiScore < 1 and epistasis > 1 (position pair 42-104, an outlier in Figure 6B, does not hold to this pattern). This suggests that the epistatic effects captured through EpiScore to active site S70 may be compensated via coupling asymmetry to all active sites, with dynamic modification of the system ultimately including both effects. A position pair that more strongly affects active site S70 via EpiScore also possesses active site-dominant coupling asymmetry and vice versa. Taken together with Figure 6B, these data indicate that dual mutants which confer less disruption to important active sites (indicated by EpiScore < 1) than their averaged individual constituents, and those which are under active site regulation, (indicated by positive %DCI_asym_) are those which display the largest degree of positive epistasis.

Thus, as a test system, TEM-1 highlights the complex relationship between mutational positions, allosteric relationships, and epistatic interplay. These emergent properties of the anisotropic residue-residue interaction network within a protein must be accounted for when attempting to fully understand or predict mutation outcomes.

### 3.3. Unidirectional Communication through DCI_asym_ Creates Cause-Effect Relationships in Allosteric Regulations

Using the dynamical picture presented above, the modulation of protein dynamics through mutations (i.e., the fluctuation response to node perturbations within a network) is similar to the modulation of dynamics through binding; this is the fundamental principle behind the concept of dynamic allostery. With the TEM-1 dual mutation positions showing unique coupling asymmetry to the active sites, it follows that there should be an obvious, unidirectional signature between allosteric sites and active sites in known allosteric proteins. Here we explore the role dynamic coupling directionality plays in allosteric regulations using an ideal model system, Pin1. Pin1 is a two-domain protein containing a catalytic PPIase domain and a distally-located WW domain, connected by a flexible (and highly disordered) interdomain linker [54,55,56]. While strictly regulated in both function and expression within healthy biological tissue [57], the up-regulation and down-regulation of Pin1 is associated with several forms of cancer and Alzheimer’s disease, respectively [57,58,59,60,61]. Studies have shown that the activity of the PPIase domain is enhanced when a ligand is bound at the non-catalytic WW domain [62,63] and communication between these two domains is requisite for proper biological function [55,64,65,66].

Previous works propose the existence of communication networks between the WW domain and the PPIase domain, including a unique allosteric pathway which only becomes active when a substrate is bound to the WW domain [62]. A further computational study indicated that pathways of communication via force propagation from the PPIase domain to the WW domain changed when a ligand was WW domain-bound [67].

**Figure 8:**
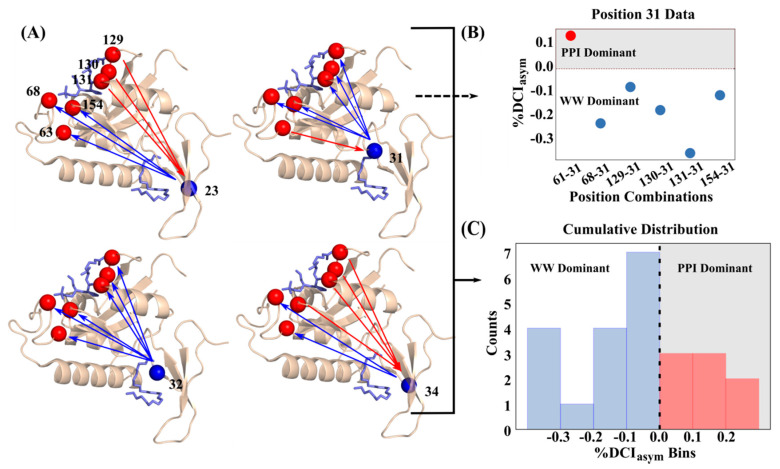
(**A**) Graphical representation of coupling asymmetry between PPIase domain binding positions (63, 68, 129, 130, 131, 154) and WW domain binding positions (23, 31, 32, 34) where blue arrows indicate the WW domain position is dominant and red arrows indicate the PPIase domain position is dominant. (**B**) Explicit values of %DCI_asym_ versus position combinations for position 31 in the WW binding domain where values above and below 0 correspond to PPIase or WW domain dominating, respectively (a value of 0 corresponds to perfect symmetry). (**C**) Full distribution of %DCI_asym_ values for all four WW domain binding positions and all six PPIase domain binding positions where 16 total residue pairs are dominated by the WW domain whereas only eight pairs are PPIase domain-dominant.

Applying our asymmetry analysis to binding pocket residues in the catalytic PPIase (%DCI_ij_) and non-catalytic WW domains (%DCI_ji_) of Pin1 (PDB ID 3TCZ [7], ligands removed), we calculate “%DCI_asym_” (%DCI_ij_ − %DCI_ji_) the coupling asymmetry between PPIase domain binding positions (63, 68, 129, 130, 131, 154) and WW domain binding positions (23, 31, 32, 34) (Figure 8). Hence, negative values indicate the WW domain position is dominant (blue arrows) whereas positive values indicate the PPIase domain position is dominant (red arrows). We see that each of the four positions in the WW domain exhibit unique asymmetric coupling with the PPIase domain positions, even when the WW domain positions are close to one another. However, with the exception of coupling between position 63 and 31, the behavior of the PPIase domain positions is unique to their catalytic loop grouping (e.g., {63,68}, {129,130,131}), where each position within a group has the same asymmetry directionality to a given WW domain position. Overall, however, the full %DCI_asym_ distribution indicates that there is a clear bias toward unidirectionality from the WW domain to the PPIase domain; the WW domain is dynamic coupling-dominant over the PPIase domain, with twice as many residue pairs exhibiting WW-dominant coupling than the reverse (16/24 vs. 8/24, Figure 8C).

This suggests a cause-and-effect relationship exists between the two domains. Using this framework, a ligand binding event is modeled as a force perturbation to the binding positions in each domain. Upon these random force perturbations, we find that, overall, the WW domain is able to induce a stronger perturbation response in the PPIase domain than the reverse. This is largely the expected relationship between an allosteric site and a catalytic site; communication between these sites should predominantly involve information transfer from the allosteric site to the catalytic site, indicating that %DCI_asym_ can capture communication directionality in allosteric systems from structural dynamics encoded within a given set of atomic coordinates.

## 4. Conclusions

In this work we showed how the anisotropic interaction network within a protein captures two essential emergent properties of protein evolution—epistasis and communication directionality—using the information stored in structural dynamics alone. Additionally, EpiScore can capture the behavior of dual-mutation epistatic outcomes with some consistent trends across different protein systems. As seen in pfDHFR, mutation pairs with a lower pairwise dynamic coupling versus average of individual couplings (EpiScore < 1) to FG loop positions are favorable, as dual mutations at these positions may be less likely to disrupt the FG loop’s interaction with the functionally critical M20 loop. A similar trend was also observed in the EpiScore analysis of TEM-1 dual mutants, where lower EpiScore to active site S70 was generally associated with higher positive experimental epistasis (R = −0.71) Further, the system-wide EpiScore analysis of GB1 dual mutants has shown that the position pairs with average EpiScore values > 1 were associated more frequently with negative epistasis, indicating that these positions might ultimately be more disruptive to the entire protein when mutated together. Furthermore, when dynamic coupling asymmetry analysis was applied via %DCI_asym_ to TEM-1, we found that EpiScore and epistasis both relate to dynamic coupling asymmetry, where position pairs which exhibited high EpiScores associated with negative epistasis also exhibited mutation-dominant coupling asymmetry. This suggests that %DCI_asym_ and EpiScore may both capture factors which contribute towards the biochemical outcome of dual mutations. If both mutational sites dominate the dynamics coupling with the active site (i.e., the active site responds more to mutational site perturbations), then dual mutations on both sites lead to negative epistasis.

As modulation of normal modes and protein dynamics is not only a tool used in evolution but also a principle exploited via allostery, we used an “ideal” allosteric system, Pin1, and observed the dynamic coupling asymmetry between a well-identified allosteric domain and an enzymatically active domain exhibits behavior that, as expected, showed the allosteric WW domain to dominate communication to the PPIase domain. Overall, these two novel protein dynamics-based metrics provide steps to mechanistically describe these complicated interactions, and also shed light on the complex anisotropic interaction network which ultimately gives rise to epistasis and allosteric regulation. They can be useful to predict mutational outcomes, particularly for those sites distal from the active site that can modulate function [68].
